# CNTN2 Deficiency Disrupts Ascending Auditory Neuroglial Interactions and Redox Homeostasis in Neural Presbycusis

**DOI:** 10.1002/advs.202522800

**Published:** 2026-10-26

**Authors:** Penghui Chen, Jingyi Zhu, Sihan Huang, Beibei Xu, Maoli Duan, Jingrong Lv, Jiarui Chen, Jun Yang, Shule Hou

**Affiliations:** ^1^ Department of Otorhinolaryngology‐Head and Neck Surgery Xinhua Hospital Shanghai Jiao Tong University School of Medicine Shanghai China; ^2^ Department of Otorhinolaryngology‐Head and Neck Surgery Shanghai Children's Hospital Shanghai Jiao Tong University School of Medicine Shanghai China; ^3^ Ear Institute Shanghai Jiao Tong University School of Medicine Shanghai China; ^4^ Ear Nose and Throat Patient Area, Trauma and Reparative Medicine Theme Karolinska University Hospital Stockholm Sweden; ^5^ Division of Ear, Nose and Throat Diseases Department of Clinical Science, Intervention and Technology Karolinska Institutet Stockholm Sweden

**Keywords:** ascending auditory pathway, Cntn2, neural presbycusis, neuroglial interactions, redox imbalance

## Abstract

Neural presbycusis disrupts auditory timing and speech perception, yet its molecular basis remains poorly understood. This study identifies *Cntn2* as the Ranvier's nodal molecule significantly downregulated in aged auditory nerves. *Cntn2* heterozygous knockout (*Cntn2^+/−^*) mice showed elevated auditory brainstem response thresholds and prolonged wave latencies and interpeak intervals, whereas distortion product otoacoustic emission thresholds were largely preserved. Hair cell and ribbon synapses were preserved, with no obvious ultrastructural changes, suggesting that overt hair cell loss or synaptic structural loss is unlikely to be the main cause of the early phenotype. Instead, transmission electron microscopy revealed myelin outfoldings and aberrant node of Ranvier morphology in myelinated auditory nerve fibers, accompanied by increased Schwann cell density. Multiplex immunofluorescence and Western blot analysis showed disruption of nodal molecular organization along the ascending auditory pathway from cochlea to auditory cortex. Transcriptomic profiling and oxidative stress assays further linked CNTN2 deficiency to altered neuroglial interaction and redox imbalance. Early unilateral AAV‐mediated CNTN2 restoration partially improved threshold and latency abnormalities. These findings identify CNTN2‐dependent neuroglial integrity as an important contributor to auditory conduction of neural presbycusis and may provide a new direction for future therapeutic studies.

## Graphical abstract

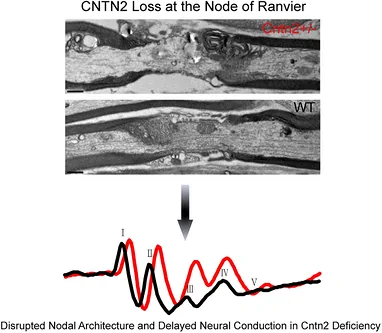

## 1 Introduction

Presbycusis or Age‐related hearing loss (ARHL) affects over one third of adults aged 65 and older [1, 2]. Neural presbycusis represents a particularly debilitating subtype characterized by impaired speech recognition that cannot be explained by threshold elevation alone [3, 4, 5]. Clinically, this phenotype resembles auditory neuropathy or hidden hearing loss (HHL) [6, 7]. No effective treatments are currently available [8, 9].

In mammals, the ascending auditory pathway originates in the organ of Corti within the cochlea [10, 11]. Inner hair cells (IHCs) perform mechanoelectrical transduction, activating spiral ganglion neurons (SGNs) of the auditory nerve (AN) [12, 13]. These neurons relay auditory information through sequential brainstem nuclei, including the cochlear nucleus and superior olivary complex, ultimately reaching the tonotopically organized auditory cortex [14, 15]. Along the ascending auditory pathway, neurons interact closely with glial cells [16, 17]. These neuroglial interactions maintain myelin, axonal domains, and nodal/ paranodal/ juxta‐paranodal molecular organization [18, 19, 20, 21]. The node of Ranvier enables rapid saltatory conduction and contains distinct molecular domains: nodal sodium‐channel complexes, paranodal adhesion complexes including CNTN1/CNTNAP1/NFASC155, and juxta‐paranodal complexes containing CNTN2, CNTNAP2, and potassium channels [22, 23, 24]. Disruption of these structures can slow conduction and impair neural synchrony [25].

Previous work has implicated myelin degeneration and nodal disorganization in ARHL and other auditory neuropathy‐like disorders [26, 27, 28, 29]. To investigate age‐related changes in neuroglial interactions within the AN, we analyzed publicly available datasets for differentially expressed genes (DEGs) between young and aged mice. Among the findings, Cntn2 expression was reduced by 55.8% in aged mice [30]. *Cntn2* encodes Cntn2, a neuronal adhesion molecule belonging to the immunoglobulin superfamily (IgSF) [31]. Cntn2 plays a key role in axonal growth, fasciculation, neuronal migration, and nerve repair and is involved in myelin formation by interacting with other IgSF members (particularly L1, Cntnap2, and NrCAM) to form the juxta‐paranodal region of the node of Ranvier [32]. We therefore combined aging cohort analysis, Cntn2 heterozygous mice, ABR, and DPOAE testing, cochlear synapse and myelin morphology, nodal molecule immunostaining, and Western blot validation, transcriptomics, oxidative stress assays, and AAV‐mediated rescue (Figure S2). Our findings may provide new theoretical insights and help identify potential therapeutic targets for the clinical management of neural presbycusis.

## 2 Results

### 2.1 Age‐Related Decline in Auditory Neuroglial Interaction Molecules

To explore the molecular basis of age‐related changes in the auditory system, we conducted a preliminary analysis of differentially expressed genes (DEGs) in the cochlear AN between young and aged mice using publicly available RNA sequencing datasets from the NCBI Gene Expression Omnibus (GSE133823, GSE141865) [29]. This analysis focused on a curated list of genes related to nodes of Ranvier, including voltage‐gated ion channels and cell adhesion molecules. The mRNA expression levels of several nodal components: *Nrcam2*, *Nrcam*, *Cntn1*, *Cntnap2*, *Nrcam1*, *Kcna2*, *Scn8a*, *L1cam*, *Cntn2*, and *Cntnap1* were significantly reduced in the cochlear AN of aged mice (Figure 1A). Notably, *Cntn2* expression exhibited a 55.8% decrease in aged mice compared with young controls (*p* < 0.01; Figure 1Axi).

**FIGURE 1:**
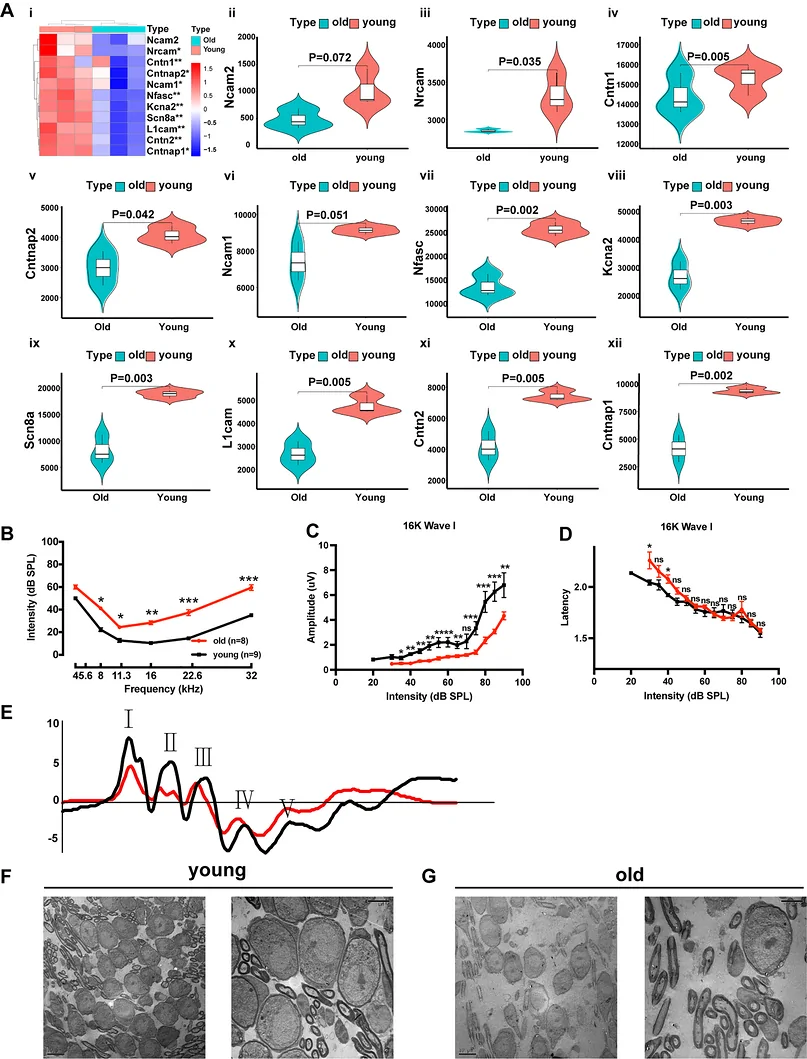
Age‐related auditory dysfunction and progressive decline of nodal adhesion molecules in the auditory nerve of aging mice. (A) Bioinformatic analysis of publicly available RNA sequencing datasets (GSE133823, GSE141865) from young and aged CBA/CaJ mice. *(i)* Heat map of differentially expressed genes (DEGs) encoding nodal microdomain molecules. *(ii–xii)* Violin plots of individual gene expression levels. *Cntn2* mRNA is reduced by 55.8% in aged mice (*p* < 0.01). Data are mean ± SD, Student's two‐tailed *t*‐test. (B)Auditory brainstem response (ABR) hearing thresholds in young (2‐month‐old, *n* = 9) and aged (12‐month‐old, *n* = 8) CBA/CaJ mice. Aged mice exhibit elevated thresholds across frequencies (4–32 kHz), indicating progressive hearing loss. (C, D) Wave I amplitude and latency at 16 kHz. Aged mice show reduced amplitude and prolonged latency. (E) Representative ABR waveforms at 80 dB SPL, in young (black) and aged (red) mice, showing delayed peak latencies and reduced amplitudes in aged mice. Wave peaks I–V are indicated. (F, G) Transmission electron micrographs (TEM) of SGNs in young (F) vs. aged (G) CBA/CaJ mice. Aged mice show fewer SGNs and thinner myelin sheaths with lamellar disorganization, indicating demyelination changes. Scale bars: 10 & 5 µm. Data are mean ± SD. ABR thresholds across frequencies were analyzed using two‐way repeated‐measures ANOVA followed by Bonferroni's multiple‐comparisons test. Single‐outcome comparisons were analyzed using two‐sided unpaired Student's t‐tests. *p < 0.05, **p < 0.01.

Compared with young mice (2 months old), aged mice (12 months old) exhibited elevated ABR thresholds across frequencies, and significantly prolonged latencies and reduced amplitudes at 16 kHz (Figure 1B–E). TEM analysis revealed signs of demyelination around SGNs in the cochlea of aged mice, including thinning and degeneration of the myelin sheath surrounding axons, increased SGNs loss, and reduced SGNs density compared to young controls (Figure 1F,G).

To validate cross‐strain conservation and exclude strain‐specific artifacts, we directly compared C57BL/6J and CBA/CaJ mice at 2 months of age. ABR thresholds, wave I amplitudes, and latencies at 16 kHz were comparable between the two strains (Figure S3A–C), confirming similar auditory baselines in young adulthood. We next examined age related changes in C57BL/6J mice. Aged C57BL/6J mice (12 months) exhibited elevated ABR thresholds at high frequencies and reduced wave I–II amplitudes with prolonged latencies at 16 kHz compared to young controls (Figure S3D–H). Notably, however, the interpeak interval (II–I) remained largely unchanged in aged C57BL/6J mice (Figure S3I), contrasting with the marked IPI prolongation observed in *Cntn2* ^+/−^ mice (Figure 3).

Next, we performed immunostaining using antibodies against CNTN2, CNTN1, NRCAM, Nav1.6, and Kv1.2. Results revealed that the protein expression levels of these nodal components deteriorated with age (Figure 2). These structural proteins, which are essential for neuroglial interactions at the nodes of Ranvier, were significantly reduced at the mRNA and protein levels in aged C57BL/6J mice. These findings suggest that neuroglial interaction dysfunction may play a critical role in neural presbycusis pathogenesis.

**FIGURE 2:**
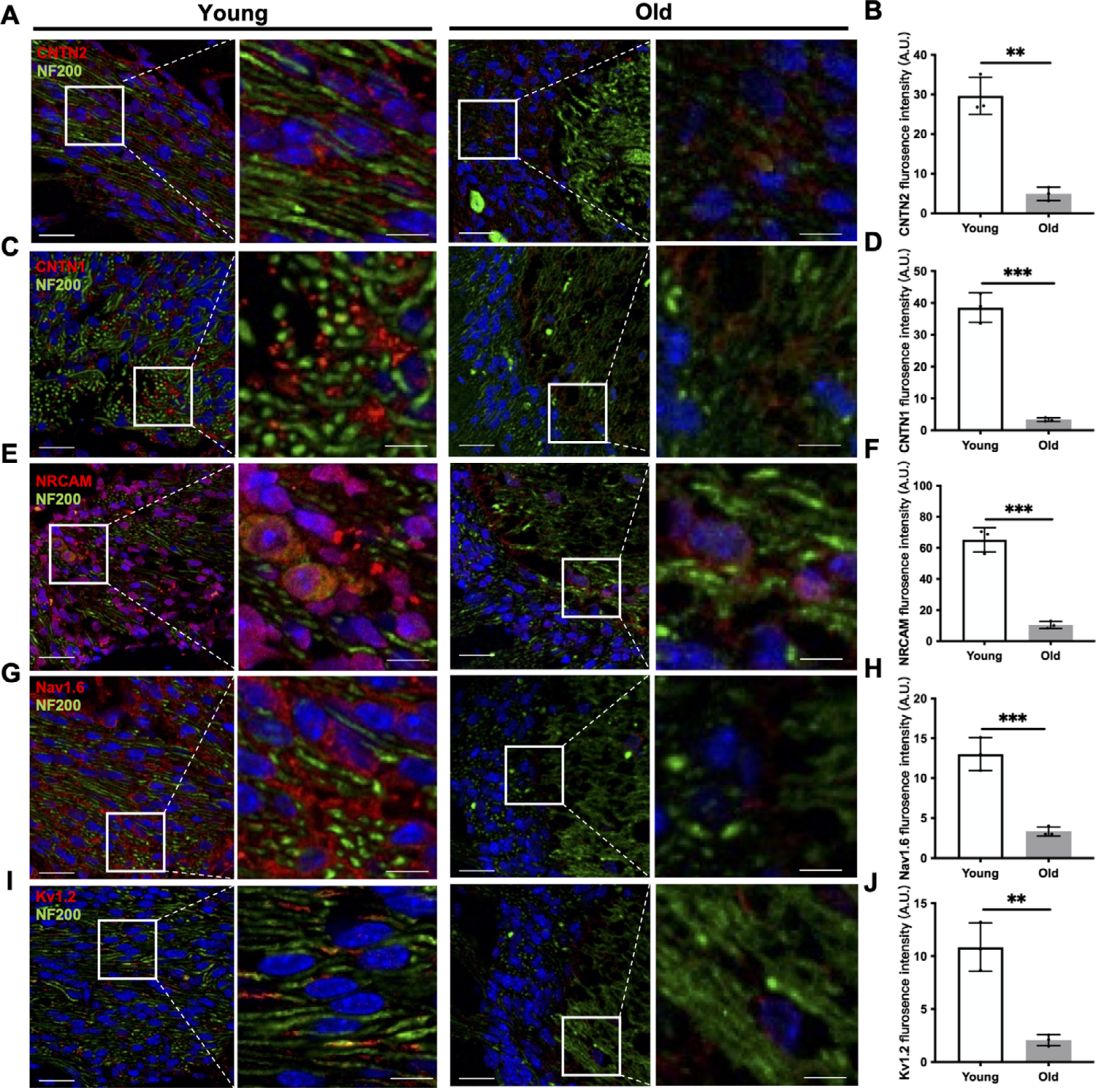
Progressive downregulation of node‐related proteins in the auditory nerve of aged mice. (A, C, E, G, I) Immunofluorescence staining of CNTN2, CNTN1, NRCAM, Nav1.6, and Kv1.2 (red) in cochlear auditory nerve sections from young (2‐month‐old) and aged (12‐month‐old) C57BL/6J mice. Green: NF200 (neurofilament, nerve fiber marker); blue: DAPI (nuclei). Insets show high‐magnification views of nodal regions. Scale bars: 20 µm (main panels) and 5 µm (insets). (B, D, F, H, J) Quantitative analysis of fluorescence intensity for each nodal marker in SGNs and nerve fibers. All five proteins show significant age‐related reduction. Data are mean ± SD; **p < 0.01, ***p < 0.001; Student's two tailed t‐test; n = 3 mice per group.

### 2.2 Cntn2 Deficiency Causes Neural Predominant Auditory Dysfunction With Preserved DPOAE Thresholds

To define the auditory phenotype caused by CNTN2 deficiency, we examined 2‐month‐old *Cntn2* ^+/−^ mice and wild‐type (WT) littermates on the same C57BL/6J background. *Cntn2* ^+/−^ mice showed elevated ABR thresholds at 4, 8, 11, 16, and 22.6 kHz, whereas the difference at 32 kHz was not significant in the threshold plot (Figure 3A). Representative ABR waveforms showed delayed peaks in *Cntn2* ^+/−^ mice (Figure 3B).

**FIGURE 3:**
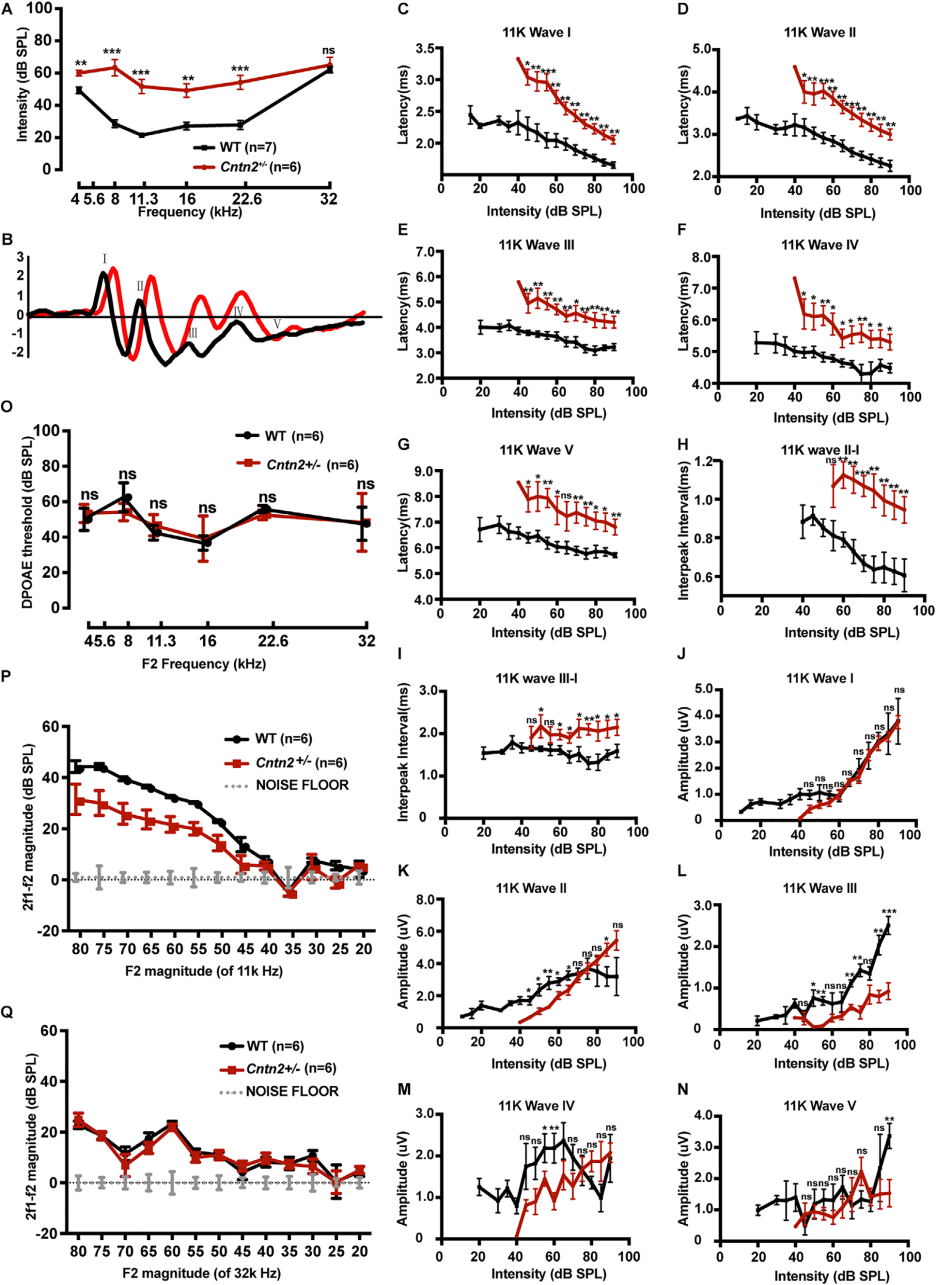
Cntn2 deficiency causes a neural‐predominant auditory dysfunction with largely preserved outer hair cell function in 2‐month‐old mice. (A) ABR hearing thresholds in 2‐month‐old *Cntn2* ^+/−^ mice (*n* = 6) and wild‐type (WT) littermate controls (*n* = 7) on the C57BL/6J background. *Cntn2* ^+/−^ mice exhibit elevated thresholds at 4, 8, 11, 16, and 22 kHz; the difference at 32 kHz is not statistically significant. (B) Representative ABR waveforms at 11 kHz, 80 dB SPL, showing delayed peak latencies (waves I‐V indicated) in *Cntn2^+/−^* mice (red) vs. WT (black). (C–I) Latency intensity functions and interpeak intervals (IPIs) for waves I–V at 11 kHz. *Cntn2* ^+/−^ mice display significantly prolonged latencies for waves I–V and increased IPIs (II‐I, III‐I). (J–N) Amplitude intensity functions for ABR waves I‐V at 11 kHz. Wave I amplitude is comparable; higher order waves show variable reductions. (O) Distortion product otoacoustic emission (DPOAE) thresholds showing no significant differences between genotypes. (P, Q) DP‐gram input‐output functions at 11 and 32 kHz. At 11 kHz, *Cntn2^+/−^* mice show modestly reduced 2f1‐f2 magnitudes at higher stimulus levels, whereas 32 kHz responses are comparable, supporting largely preserved OHC function. Data are mean ± SD. ABR and DPOAE measurements were analyzed using two‐way repeated‐measures ANOVA with Bonferroni's multiple‐ comparisons test. ns, *p* > 0.05, *p < 0.05, **p < 0.01, ***p < 0.001.

At 11 kHz, *Cntn2* ^+/−^ mice displayed prolonged latencies of ABR waves I‐V over a broad range of stimulus intensities, together with increased interpeak intervals II‐I and III‐I (Figure 3C–I). In contrast, wave I amplitude was not significantly different between groups, and higher‐order wave amplitudes showed frequency and intensity‐dependent changes (Figure 3J–N). At 32 kHz, *Cntn2* ^+/−^ mice also exhibited prolonged latencies for waves I‐V and increased IPIs (II‐I, III‐I, IV‐I, V‐I, V‐III), while wave amplitudes showed more variable differences (Figure S4).

To test whether the ABR phenotype could be explained by outer hair cell (OHC) dysfunction, we added DPOAE measurements. DPOAE thresholds across tested F2 frequencies did not differ significantly between *Cntn2* ^+/−^ and WT mice (Figure 3O), and DP‐gram analysis at 32 kHz showed comparable 2f1‐f2 magnitudes (Figure 3Q). At 11 kHz, *Cntn2* ^+/−^ mice showed reduced 2f1‐f2 magnitudes at higher stimulus levels (Figure 3P), indicating that OHC function is largely preserved but not uniformly identical across all stimulus conditions. Together, these results support a neural‐predominant auditory phenotype rather than a primary sensory hair cell lesion.

### 2.3 Cntn2 Deficiency Induced Auditory Neuropathy is not Caused by Synapse Loss

One known mechanism of auditory neuropathy involves synaptic dysfunction at IHCs [33, 34, 35]. However, *Cntn2* ^+/−^ mice exhibited no gross morphological abnormalities in the cochlea (Figure 4A–C). To assess individual synaptic pairing directly, we examined the original confocal Z‐stacks containing separate CtBP2 and GluR2 channels. CtBP2‐positive puncta juxtaposed to GluR2‐positive puncta were counted as paired ribbon synapses. The number of paired CtBP2/GluR2 puncta per IHC did not differ significantly between WT and *Cntn2* ^+/−^ mice (Figure 4D,E). Serial TEM of ribbon synapses further showed no significant difference in ribbon associated synaptic vesicles or ribbon diameter between *Cntn2* ^+/−^ and WT mice (Figure 4F–H). These data indicate that the early CNTN2 deficient phenotype is not attributable to overt hair cell loss or detectable loss of ribbon synapses.

**FIGURE 4:**
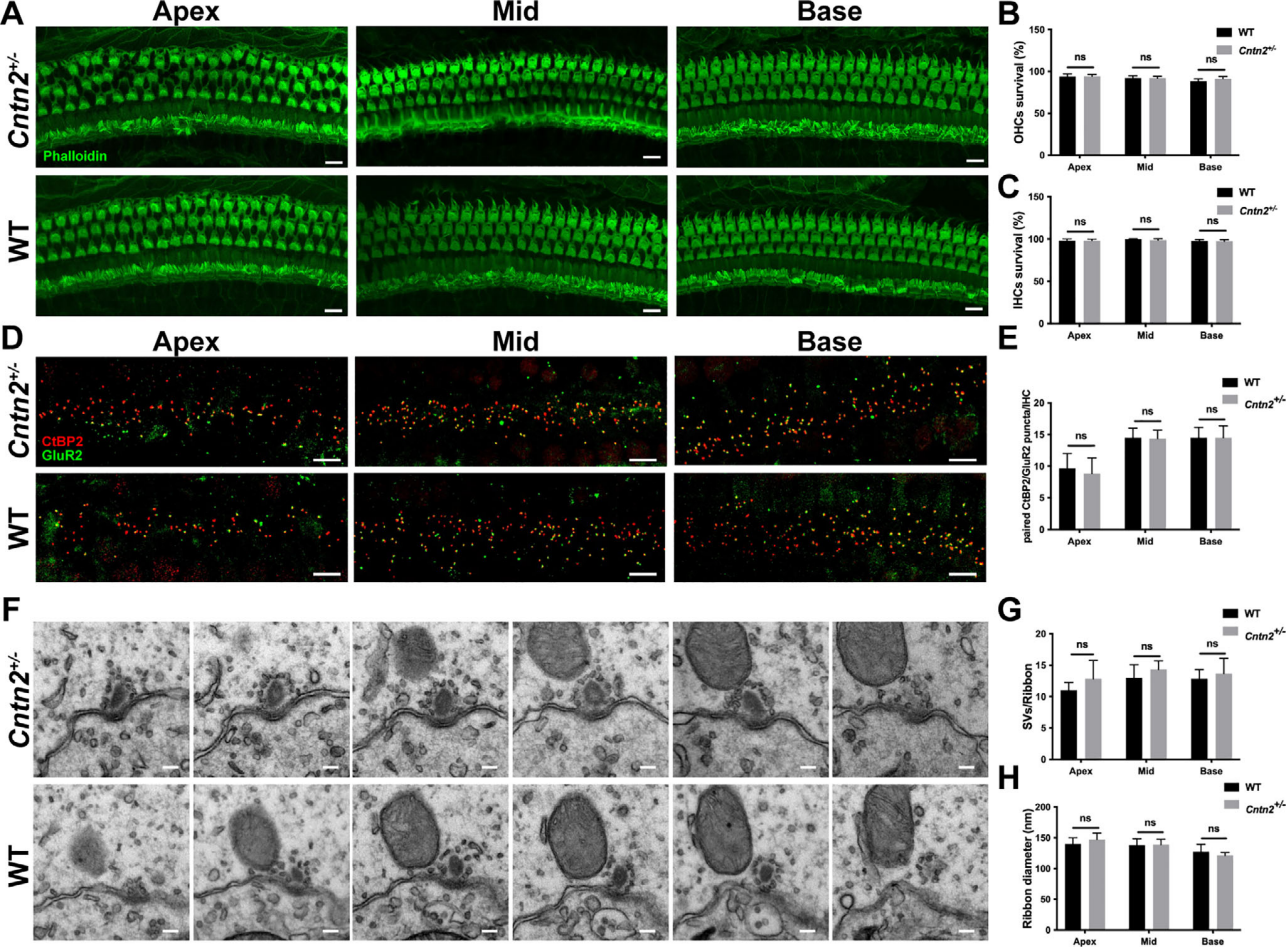
Preservation of cochlear hair cells and ribbon synaptic architecture in Cntn2‐deficient mice. (A) Whole‐mounting phalloidin staining of the organ of Corti showing intact three rows of outer hair cells (OHCs) and one row of inner hair cells (IHCs) across apical, middle, and basal turns in 2‐month‐old WT and *Cntn2* ^+/−^ mice. Scale bar: 10 µm. (B, C) Quantification of OHC and IHC survival percentages; no significant differences. (D) Representative CtBP2‐positive presynaptic ribbons (red) and GluR2‐positive postsynaptic puncta (green) in the IHC region. A CtBP2‐positive punctum juxtaposed to a GluR2‐positive punctum in the Z‐stack was counted as one paired ribbon synapse. Scale bar: 7.5 µm. (E) Quantification of paired CtBP2/GluR2 puncta per IHC in the apical, middle, and basal cochlear regions. (F) Representative serial transmission electron micrographs (70 nm ultrathin sections) through IHC ribbon synapses. Scale bar: 500 nm. (G, H) Quantification of synaptic vesicle number per ribbon and ribbon diameter; no significant differences. For B, C, and E, two‐way repeated‐measures ANOVA followed by Bonferroni's multiple‐comparisons test was used. For G and H, two‐sided unpaired Student's t‐tests were used. Data are mean ± SD; ns *p* > 0.05. *n* = 3 mice per group.

### 2.4 Cntn2 Deficiency Disrupts Myelin and Nodal Organization in the Auditory Nerve

TEM was used to examine the ultrastructural morphology of cochlear ANs in *Cntn2* ^+/−^ and WT mice. TEM revealed prominent abnormalities in *Cntn2* ^+/−^ mice, including myelin outfoldings, loosened lamellae, and aberrant lamellar folding in myelinated auditory nerve fibers (Figure 5A). Higher magnification views revealed disorganized myelin lamellae with paranodal detachment (Figure 5A). Quantification confirmed a significantly increased percentage of myelinated axons exhibiting myelin outfoldings (Figure 5C). In addition to myelin abnormalities, a significant increase in Schwann cell density was observed in *Cntn2* ^+/−^ mice (Figure 5B,D). Quantitative morphometry showed no significant genotype differences in mean myelin lamellar number or myelin thickness (Figure S5). Longitudinal TEM sections through nodes of Ranvier (N) revealed that *Cntn2* ^+/−^ mice exhibit redundant myelin looping, lamellar dissociation, and terminal loop detachment from the axolemma within the juxta‐paranodal (JXP) and paranodal (Pn) regions, indicating aberrant node of Ranvier morphology (Figure 5E). Collectively, these findings highlight the essential role of Cntn2 in maintaining neuroglial integrity, particularly in the stabilization and assembly of myelin sheaths and nodal structures in myelinated auditory nerve fibers.

**FIGURE 5:**
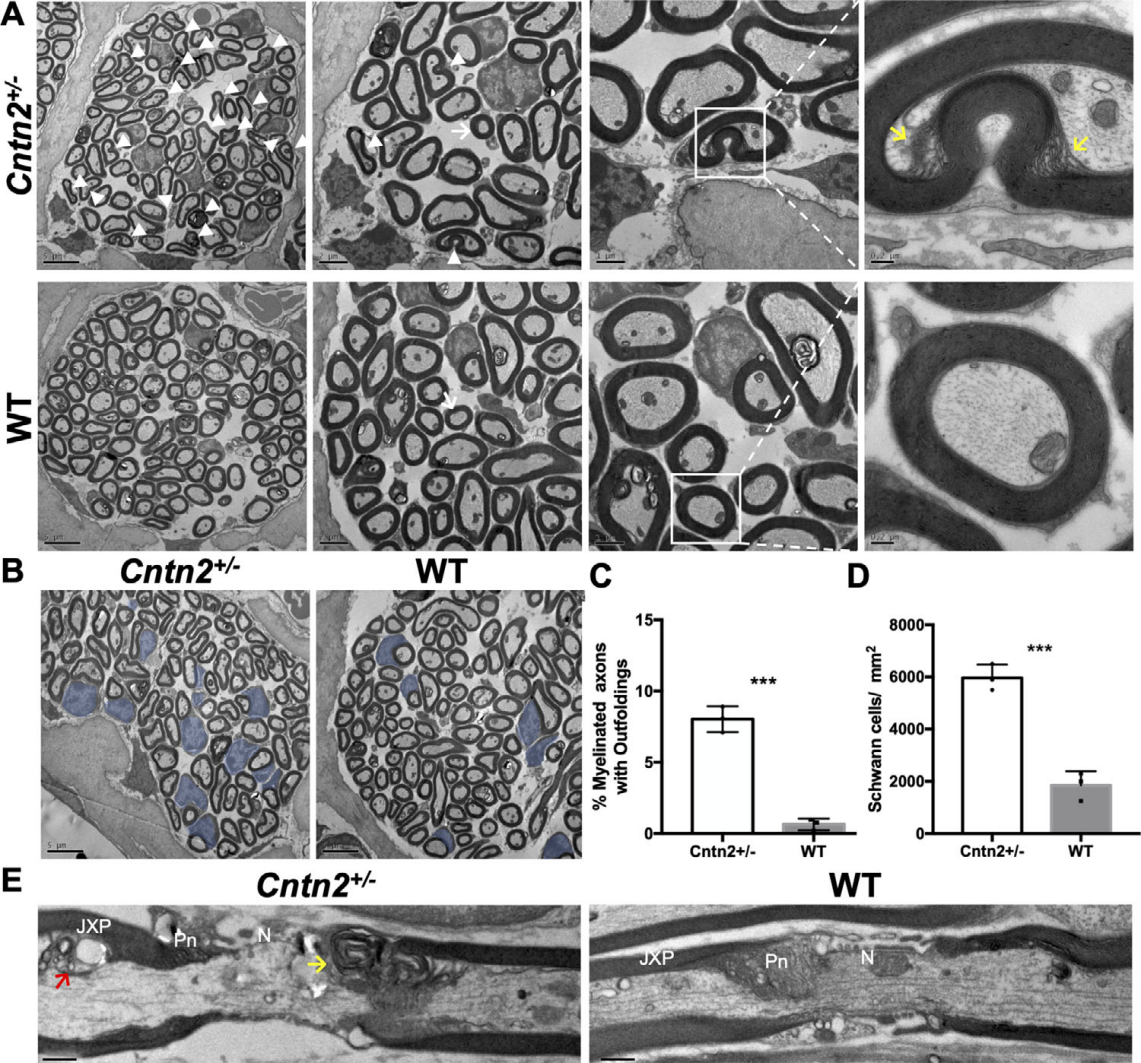
Cntn2 deficiency induces myelin outfoldings, increased Schwann cell density, and nodal disorganization in the auditory nerve. (A) Transverse TEM sections of cochlear auditory nerve (AN) in 2‐month‐old *Cntn2^+/−^* (upper row) and WT littermate (lower row) mice. Thick white arrows indicate myelin outfoldings in large‐caliber myelinated fibers; thin white arrows indicate relatively preserved small‐caliber myelinated fibers; yellow arrows indicate loosened and fragmented myelin lamellae. Higher magnification views (rightmost panels) reveal disorganized myelin lamellae with paranodal detachment in *Cntn2^+/−^* vs. compact, regularly layered myelin in WT. Scale bars: 5, 2, 1 and 0.2 µm. (B) Low magnification transverse views of AN fascicles with blue pseudocolor highlighting Schwann cells, showing increased endoneurial cellularity in *Cntn2^+/−^* mice. Scale bar: 5 µm. (C) Quantification of the percentage of myelinated axons exhibiting myelin outfoldings. (D) Quantification of Schwann cell density per mm^2^. (E) Longitudinal TEM sections through nodes of Ranvier (N) in Type I fibers. JXP, juxtaparanode; Pn, paranode. *Cntn2^+/−^* mice (left) show redundant myelin looping (yellow arrows in E), lamellar dissociation, and terminal loop detachment from the axolemma (red arrows), indicating aberrant node of Ranvier morphology. WT (right) shows tightly apposed terminal loops forming regular axo‐glial junctions (arrowheads). Scale bar: 500 nm. Data are mean ± SD; ***p < 0.001; Student's two‐tailed t‐test; n = 3 mice per group.

### 2.5 Cntn2 Deficiency Disrupts Nodal Microdomain Organization Along the Ascending Auditory Pathway

ANs transmit auditory information from the organ of Corti to the auditory cortex via multiple brainstem and midbrain nuclei [36]. Along the ascending auditory pathway, glial cells closely interact with neurons to support precise and rapid signal conduction [37]. These interactions involve myelin formation and molecular microdomain assembly that constitutes the node of Ranvier. This node comprises three distinct microdomains: the node, containing cell adhesion molecules such as NRCAM; the paranode, which includes Cntn1; and the juxta‐paranode, composed of Cntn2, and the voltage‐gated potassium channel Kv1.2 [38].

To assess neuroglial interactions and the structural integrity of the nodes of Ranvier following Cntn2 deficiency, we performed immunofluorescence staining and confocal imaging for Kv1.2, Cntn2, Cntn1, and NRCAM in the cochlear SGNs (Figure 6), cochlear nucleus (Figure 7A–E), and auditory cortex (Figure 7F–J) of *Cntn2* ^+/−^ and WT mice. In *Cntn2* ^+/−^ mice, expression levels of Kv1.2, Cntn2, Cntn1, and NRCAM were significantly reduced in cochlear SGNs, indicating varying degrees of disruption in the juxta‐paranodal, paranodal, and nodal microdomains. Multiplex immunofluorescence staining further demonstrated that these proteins were also significantly downregulated in both the cochlear nucleus and auditory cortex of *Cntn2* ^+/−^ mice. Western blot analysis validated these immunofluorescence findings. CNTN2, CNTN1, Kv1.2, and NRCAM were significantly reduced in cochlear auditory nerve lysates from *Cntn2* ^+/−^ mice (Figure S6). These proteins were also downregulated in the cochlear nucleus and auditory cortex (Figure S7). These findings suggest that Cntn2 deficiency leads to widespread structural damage to the nodes of Ranvier along the entire ascending auditory pathway, impairing neuroglial interaction, and potentially contributing to auditory dysfunction.

**FIGURE 6:**
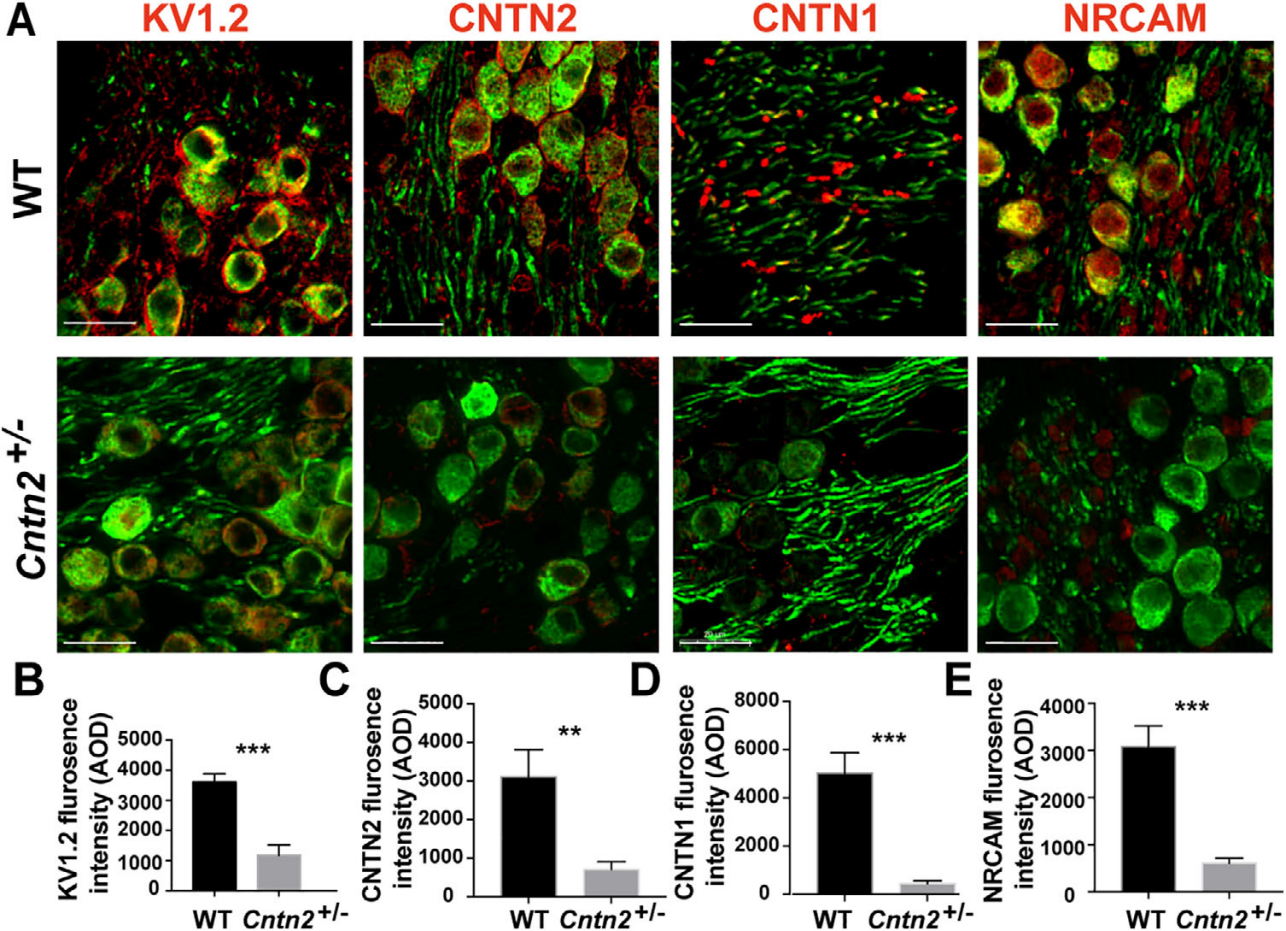
Disruption of nodal microdomain organization in cochlear spiral ganglion neurons of Cntn2‐deficient mice. (A) Immunofluorescence staining of cochlear spiral ganglion neurons (SGNs) for juxta‐paranodal and nodal proteins: Kv1.2, CNTN2, CNTN1, and NRCAM (red). Green: NF200 (nerve fibers); blue: DAPI (nuclei). In *Cntn2* ^+/−^ mice, all four proteins show markedly reduced expression and disrupted localization compared to WT littermates. Scale bar: 10 µm. (B–E) Quantitative analysis of fluorescence intensity for Kv1.2 (B), CNTN2 (C), CNTN1 (D), and NRCAM (E). Data are mean ± SD; **p < 0.01, ***p < 0.001; Student's two‐tailed *t*‐test; *n* = 3 mice per group.

**FIGURE 7:**
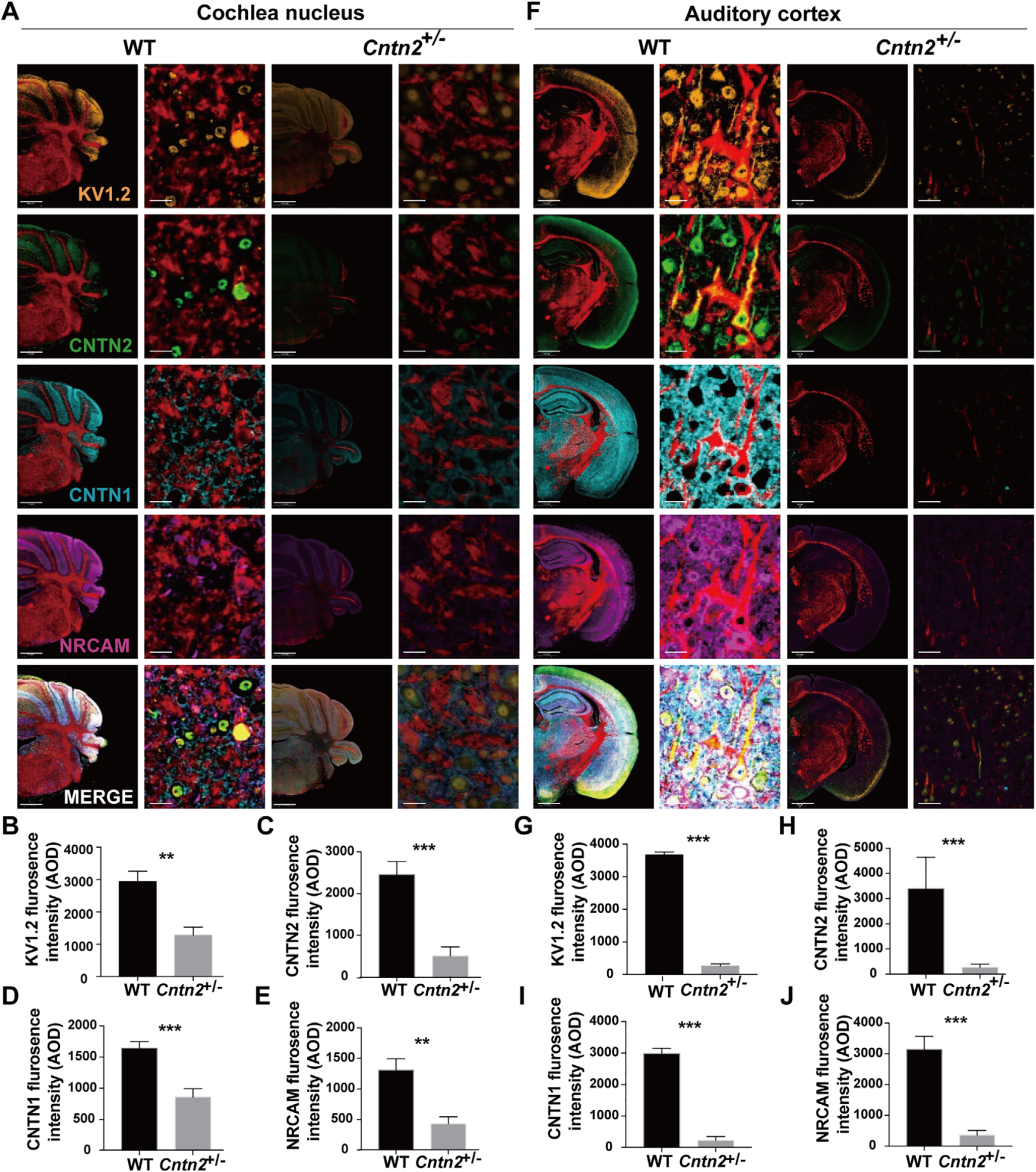
Widespread downregulation of nodal proteins along the ascending auditory pathway in Cntn2‐deficient mice. (A) Multiplex immunofluorescence staining of microdomain molecules in the cochlear nucleus (left panels) and auditory cortex (right panels). Orange: Kv1.2; green: CNTN2; cyan: CNTN1; purple: NRCAM; red: NF200. Low magnification views show anatomical localization; high magnification insets reveal protein expression at nodal structures. Scale bars: 1000 µm (low magnification) and 20 µm (high magnification). (B–E) Quantification in the cochlear nucleus: Kv1.2 (B), CNTN2 (C), CNTN1 (D), and NRCAM (E). (G–J) Quantification in the auditory cortex: Kv1.2 (G), CNTN2 (H), CNTN1 (I), and NRCAM (J). All proteins are significantly reduced in *Cntn2* ^+/−^ mice. Data are mean ± SD; **p < 0.01, ***p < 0.001; Student's two‐tailed *t*‐test; *n* = 3 mice per group.

Consistent with the prolonged latencies and IPIs observed across all ABR waves, immunofluorescence analysis revealed that Cntn2 deficiency leads to neuroglial interaction dysfunction, and structural disruption of the nodes of Ranvier at every stage of the ascending auditory pathway.

### 2.6 Cntn2 Deficiency Alters Neuroglial Interaction and Oxidative Stress Pathways

To investigate the molecular mechanisms underlying AN demyelination in *Cntn2* ^+/−^ mice, we conducted transcriptome analysis using RNA extracted from the ANs of 2‐month‐old *Cntn2* ^+/−^ and WT mice. DEGs were defined by a fold change threshold of >1.5 and a *P* <0.05. To explore the biological significance of the DEGs, gene ontology (GO) enrichment analysis was performed. GO terms were enriched across biological process, cellular component, and molecular function categories, including regulation of neuron projection development, sensory perception of sound, axonogenesis, Schwann cell development, regulation of axon diameter, glial cell differentiation, axon structure, glial cell projection, oxidoreductase activity, and peroxidase activity (Figure 8A). These enriched terms are likely associated with the mechanisms of hearing loss observed in *Cntn2* ^+/−^ mice. Additionally, gene set enrichment analysis (GSEA) revealed that DEGs were significantly involved in pathways such as regulation of axonogenesis, axon initial segment formation, NCAM signaling for neurite outgrowth, ErbB signaling, oxidation by cytochrome P450, and chemical carcinogenesis–reactive oxygen species (ROS) pathways (Figure 8B–G). These transcriptomic findings indicate that Cntn2 deficiency contributes to hearing impairment by disrupting neuroglial interactions and promoting oxidative stress through dysregulation of genes involved in axonal function, glial biology, and redox homeostasis.

**FIGURE 8:**
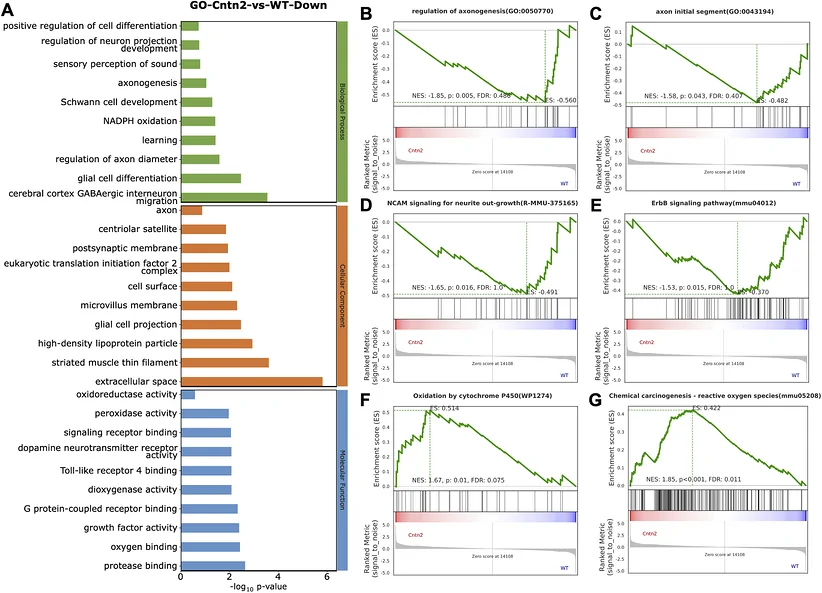
Transcriptomic profiling reveals disruption of neuroglial interactions and activation of oxidative stress pathways in Cntn2‐deficient auditory nerve. (A) Gene ontology (GO) enrichment analysis of differentially expressed genes (DEGs) across biological process, cellular component, and molecular function categories in the auditory nerve of 2‐month‐old *Cntn2^+/−^* vs. WT mice. DEGs are enriched in biological processes including “regulation of neuron projection development,” “sensory perception of sound,” “Schwann cell development,” “axon structure,” and “glial cell projection”; and in molecular functions including “oxidoreductase activity” and “peroxidase activity.” (B–E) Gene set enrichment analysis (GSEA) showing significant downregulation of pathways involved in axonogenesis (B), axon initial segment formation (C), NCAM signaling for neurite outgrowth (D), and ErbB signaling (E). (F, G) GSEA shows significant upregulation of oxidative stress‐related pathways: oxidation by cytochrome P450 (F) and chemical carcinogenesis‐reactive oxygen species (ROS) (G). Normalized enrichment scores (NES) and false discovery rates (FDR) are indicated.

### 2.7 Cntn2 Deficiency Increases Oxidative Stress Across the Auditory Pathway

To assess ROS level changes in *Cntn2^+/−^* mice, we performed 4‐hydroxynonenal (4‐HNE) and dihydroethidium (DHE) staining to evaluate oxidative stress across the auditory pathway. 4‐HNE, a highly reactive byproduct of lipid peroxidation, serves as a stable marker for oxidative stress. It forms adducts with sulfhydryl, histidine, and lysine residues, which can be visualized using specific antibodies [39]. DHE is a lipophilic fluorescent dye that, upon oxidation by superoxide radicals [40]. As expected, the fluorescence intensity of 4‐HNE (Figure 9A–D) and DHE staining (Figure 9E–H) was significantly elevated in the cochlea, cochlear nucleus, and auditory cortex of *Cntn2* ^+/−^ mice compared with WT controls. Western blot analysis of 4‐HNE levels were also significantly elevated in the cochlea, cochlear nucleus, and auditory cortex of *Cntn2^+/−^* mice (Figure S8). These results indicate that Cntn2 deficiency leads to increased oxidative stress across the ascending auditory pathway, contributing to extended cellular damage.

**FIGURE 9:**
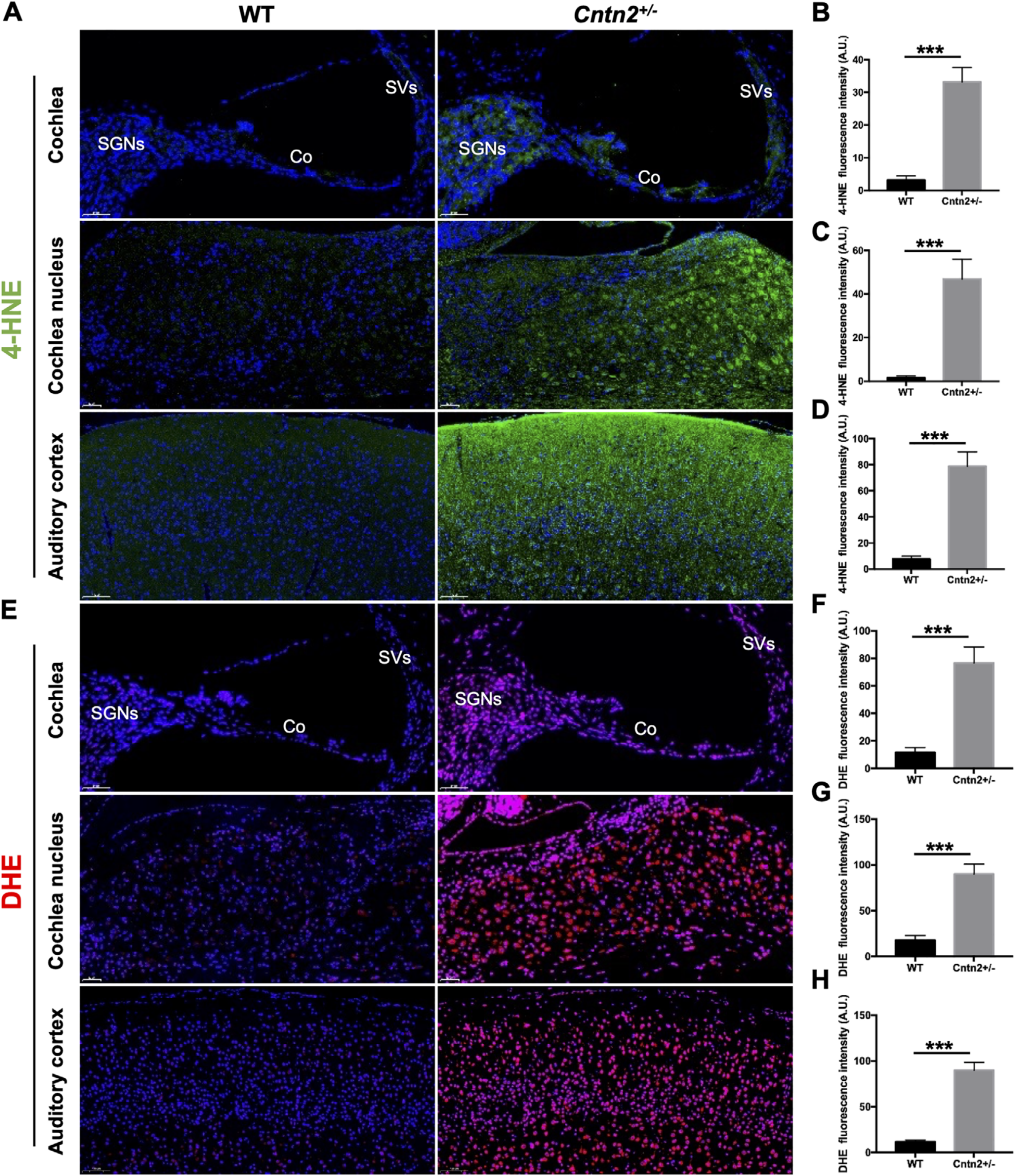
Exacerbated oxidative stress across the ascending auditory pathway in Cntn2‐deficient mice. **(A‐D)** 4‐hydroxynonenal (4‐HNE) immunofluorescence staining in the cochlea (A), cochlear nucleus (B), and auditory cortex (C, D). 4‐HNE (green) indicates lipid peroxidation; blue: DAPI. **(E–H)** Dihydroethidium (DHE) staining in the cochlea (E), cochlear nucleus (F), and auditory cortex (G, H). DHE (red) indicates superoxide free radicals; blue: DAPI. Scale bar: 20 µm. Quantification panels show significantly elevated fluorescence intensity in *Cntn2* ^+/−^ mice across all three auditory levels. Data are mean ± SD; ***p < 0.001; Student's two‐tailed *t*‐test; *n* = 3 mice per group.

### 2.8 Early AAV‐Mediated CNTN2 Supplementation Partially Attenuates Auditory Neural Dysfunction

To directly test whether CNTN2 deficiency contributes to the auditory phenotype, Cntn2^+/−^ mice received AAV9‐CMV‐Cntn2 (*Cntn2* ^+/−^ + AAV‐OE group) in the left cochlea at postnatal day 1 (P1). The right cochlea of the same mouse received equal dose of empty AAV9 vector (*Cntn2* ^+/−^ group) at the same viral titer through the same round window injection route and served as the paired vector control. AAV9‐CMV‐Cntn2 contained no fluorescent reporter owing to the packaging capacity of AAV, which precluded inclusion of both full‐length Cntn2 and EGFP in the same vector. A separate cohort received AAV9‐CMV‐EGFP to evaluate cochlear transduction, and representative EGFP fluorescence from this cohort is shown in Figure 10B.

**FIGURE 10:**
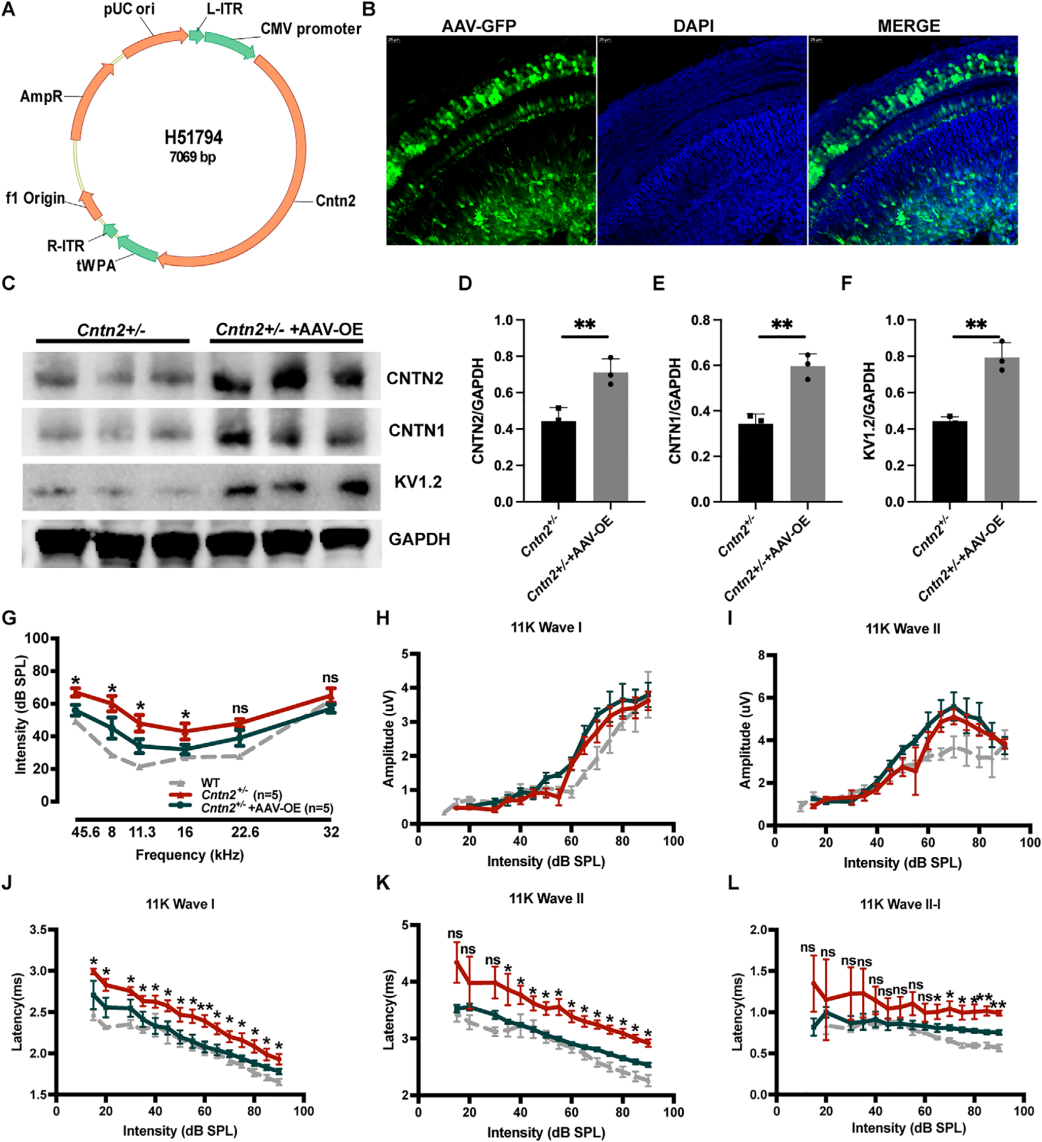
Early AAV‐mediated CNTN2 restoration partially attenuates auditory neural dysfunction in Cntn2 deficient mice. (A) Schematic of the AAV9‐CMV‐Cntn2 vector (plasmid H51794, 7069 bp), carrying full length mouse *Cntn2* cDNA driven by the CMV promoter with tWPA enhancer. (B) Representative cochlear whole mounting images showing successful AAV9 mediated transduction. EGFP fluorescence (green) confirms viral delivery to cochlear sensory epithelium and spiral ganglion neurons; blue: DAPI; right panel: merged image. Scale bar: 50 µm. (C–F) Western blot analysis of CNTN2, CNTN1, and Kv1.2 protein levels in the cochlear auditory nerve of 2‐month‐old *Cntn2* ^+/−^ mice cochlea and *Cntn2* ^+/−^ + AAV‐OE mice cochlea. GAPDH served as the loading control. Data are mean ± SD; **p < 0.01; Student's two‐tailed *t*‐test; *n* = 3 mice per group. (G) ABR hearing thresholds in WT (gray dashed line), *Cntn2* ^+/−^ group (red), and *Cntn2* ^+/−^ + AAV‐OE group (green) mice at 2 months of age. The *Cntn2* ^+/−^ + AAV‐OE group showed significant threshold reduction at 4, 8, 11, and 16 kHz compared to *Cntn2* ^+/−^; no significant difference was observed at 22.6 or 32 kHz (ns). Two‐way ANOVA with Bonferroni's multiple‐comparisons test; *n* = 5 mice per group. (H, I) Amplitude intensity functions for ABR wave I (H) and wave II (I) at 11 kHz. (J, K) Latency intensity functions for ABR wave I (J) and wave II (K) at 11 kHz. Latencies were significantly shortened in the *Cntn2* ^+/−^ + AAV‐OE group compared to *Cntn2* ^+/−^ controls. (L) Interpeak interval (IPI) for waves II‐I at 11 kHz. Data are mean ± SD. ns, *p* > 0.05; *p < 0.05; **p < 0.01; ***p < 0.001.

At 2 months of age, Western blot analysis showed higher CNTN2, CNTN1, and Kv1.2 protein levels in AAV‐OE group compared to *Cntn2* ^+/−^ controls (Figure 10C–F), indicating that CNTN2 supplementation can partially rebuild the nodal microdomain protein network.

Closed‐field ABR analysis demonstrated that the AAV‐OE‐treated group exhibited significant threshold reduction at 4, 8, 11, and 16 kHz compared to *Cntn2* ^+/−^ mice, with no significant improvement at 22.6, 32 kHz (Figure 10G). Wave I and wave II latencies at 11 kHz were significantly shortened in the AAV‐OE treated group (Figure 10J,K), and the interpeak interval (II‐I) showed partial normalization at higher stimulus intensities (Figure 10L). Wave amplitudes displayed modest recovery (Figure 10H,I).

## 3 Discussion

Neural presbycusis is usually interpreted through the loss of spiral ganglion neurons, cochlear synapses, or central auditory processing capacity, but the contribution of neuroglial integrity to auditory aging remains less well defined [41, 42]. Our findings place CNTN2 dependent nodal and paranodal organization at this interface. The reduced expression of nodal neuroglial molecules in aged auditory nerve samples, together with the phenotype of Cntn2 deficient mice, suggests that disruption of axon glia organization is closely associated with delayed auditory neural conduction. Given the different mouse strains used, these findings support a role for CNTN2 in auditory aging but do not establish a direct causal link.

The auditory phenotype in Cntn2 deficient mice is most consistent with a neural predominant form of dysfunction. ABR thresholds were elevated and wave latencies and interpeak intervals were prolonged, indicating impaired neural synchrony along the ascending auditory pathway. In contrast, DPOAE thresholds were largely preserved, and hair cell survival was not significantly reduced. These observations argue against primary outer hair cell failure as the main driver of the phenotype. The reduced 11 kHz distortion product amplitudes at high stimulus levels nevertheless suggest that cochlear sensory contribution cannot be completely excluded, and the phenotype is therefore best described as neural predominant rather than purely neural.

Cochlear synaptopathy is an import mechanism of hidden hearing loss and auditory neuropathy like phenotypes [43, 44, 45]. For this reason, we examined both presynaptic and postsynaptic synaptic structures [46, 47]. Preserved CtBP2 and GluR2 labeling, together with unchanged ribbon associated vesicles and ribbon diameter, indicates that overt ribbon synapse structural loss is unlikely to be the principal cause of the early phenotype in Cntn2 deficient mice. Instead, the present structural and functional pattern points more strongly toward impaired myelin and nodal organization as a source of delayed auditory conduction.

Although numerous studies have investigated the nodes of Ranvier in the central nervous system, research on their function within the auditory system remains limited. Auditory nerve fibers maintain rapid conduction velocity through the integrity of the myelin sheath and nodes of Ranvier [48]. Cntn2 deficient auditory nerves showed increased myelin outfoldings, lamellar disorganization, abnormal nodal morphology, and increased Schwann cell density. CNTN2 is a key component of the juxta‐paranodal region and axon‐glia adhesion. Disruption of this organization may disturb ion channel localization, paranodal barrier integrity, and axon‐glia adhesion, thereby slowing action potential propagation without requiring extensive neuronal loss [49, 50, 51]. The current data support a defect in myelinated auditory nerve fibers, but do not establish preferential involvement of a specific fiber subtype or Schwann cell proliferation.

The involvement of the ascending auditory pathway is also important. CNTN2 deficiency altered nodal and paranodal molecular organization not only in cochlear auditory nerve structures but also in central auditory nuclei and auditory cortex. This broader distribution may help explain why ABR delays were observed across multiple waves rather than being restricted to wave I [52]. In neural presbycusis, impaired speech perception often exceeds what would be predicted from threshold elevation alone [53]. A mechanism that affects temporal precision across peripheral and central auditory relays could therefore be particularly relevant to the clinical features of the disease.

Reactive oxygen species and antioxidant defense mechanisms play a critical role in presbycusis pathogenesis [54]. Transcriptome analysis revealed that differentially expressed genes in *Cntn2^+/−^* mice were enriched in pathways related to neuroglial interaction and oxidative stress. Increased 4‐HNE and DHE signals across auditory structures indicate that oxidative stress accompanies the structural changes. Whether oxidative stress is a downstream consequence of impaired axon glia interaction, a parallel response to cellular stress, or a contributor to progressive nodal instability remains to be determined. However, the convergence of nodal disruption and redox imbalance suggests that auditory neural aging may involve both structural and metabolic vulnerability.

Gene therapy for hereditary hearing loss has demonstrated remarkable efficacy in both animal studies and human clinical trials [55, 56]. Early AAV‐mediated CNTN2 supplementation partially restored nodal protein expression and improved auditory thresholds and neural timing, supporting a causal contribution of CNTN2 deficiency to the auditory phenotype. However, no significant threshold improvement was detected at 22.6 or 32 kHz, indicating incomplete functional recovery. The AAV vector was delivered through the round window membrane [57, 58, 59]. This approach achieves transduction primarily in the cochlea and spiral ganglion neurons [60]. It does not efficiently reach central auditory nuclei such as the cochlear nucleus or auditory cortex. CNTN2 expression in these central regions remained unrescued. This may limit functional recovery of higher order ABR waves. The developmental timing of paranodal assembly may create a critical window. Late restoration may not fully reverse established structural defects. Downstream oxidative stress and glial remodeling may persist even after CNTN2 replacement. These factors together likely constrained the extent of hearing recovery.

The preserved synaptic structural measures do not exclude functional abnormalities in neurotransmitter release, postsynaptic signaling, or neuronal excitability. In addition, the auditory nerve fiber subtypes affected by *Cntn2* deficiency remain unresolved. Direct electrophysiological recordings and subtype‐specific analyses will be required to define how nodal neuroglial disruption, myelin remodeling, and oxidative stress interact to impair auditory function.

Together, these findings support a model in which CNTN2‐dependent neuroglial organization contributes to the timing fidelity of the auditory pathway. Reduced CNTN2 disrupts nodal organization, myelin structure, and redox imbalance, thereby delaying auditory neural conduction. This work places neuroglial integrity as an important component of neural presbycusis and provides a basis for investigating how impaired neuroglial maintenance contributes to auditory aging.

## 4 Experimental Section

### 4.1 Animal Model

The young group comprised 2‐month‐old C57BL/6J and CBA/CaJ mice, whereas the old group comprised 12‐month‐old C57BL/6J and CBA/CaJ mice. *Cntn2* knockout mice were created using CRISPR/Cas9 mediated genome editing on the C57BL/6 background. The goal was to insert a Cre‐EGFP‐SV40 polyA cassette into exon 2 of *Cntn2*, replacing the ATG start codon to achieve *Cntn2* knockout. Heterozygous *Cntn2* mice (*Cntn2^+/−^*) were used as the experimental group, and littermate WT mice served as controls. All animals were housed in the Animal Center of Xinhua Hospital, affiliated with Shanghai Jiao Tong University School of Medicine, under controlled conditions: a 12 h light/dark cycle, temperature maintained at 22°C ± 2°C, humidity at 50% ± 10%, and access to a standard rodent diet in a ventilated caging system. All experimental procedures complied with the guidelines of the Institutional Authority for Laboratory Animal Care of Shanghai Jiao Tong University School of Medicine Affiliated Xinhua Hospital (approval number: XHEC‐NSCF‐2022‐199).

### 4.2 ABR Recording and Analysis

Mice were anesthetized with ketamine (70 mg/kg for males, 100 mg/kg for females) and medetomidine (1 mg/kg). ABRs were recorded using a Tucker‐Davis Technologies workstation in a sound‐attenuating chamber. For the aging and genotype experiments, 100 µs clicks and tone bursts at 4, 8, 11, 16, 22.6, and 32 kHz were delivered through a calibrated MF1‐M speaker under free‐field conditions. For the AAV experiment, stimuli were delivered under closed‐field conditions through a sound delivery tube inserted into the external auditory canal. The two ears were tested sequentially by repositioning the tube. Each stimulus was presented at least 512 times. Responses were amplified, filtered (0.3–3 kHz), and averaged. Hearing thresholds were determined offline as the SPL at which the first peak of wave II was visually distinguishable. Amplitude measurement: Wave amplitude was calculated using the peak‐to‐trough method, defined as the vertical distance from the wave peak to the subsequent trough. Amplitudes and latencies of waves I‐V were measured when the corresponding peaks were identifiable. Latency measurement: Latency was defined as the time from stimulus onset to the peak of each wave. Latencies of waves I, III, and V were recorded. Interpeak latency measurement: Interpeak latencies were calculated as the time intervals between wave peaks: from wave I to wave II, wave I to wave III, and wave I to wave V.

### 4.3 DPOAE Recording

DPOAEs were recorded to evaluate outer hair cell function. Two primary tones (f1 and f2) were presented at an f2/f1 ratio of approximately 1.2, and 2f1‐f2 distortion products were measured across tested F2 frequencies. DPOAE thresholds and DP‐gram magnitudes were compared between WT and *Cntn2^+/−^* mice. The noise floor was recorded and plotted for DP‐gram analysis.

### 4.4 Immunofluorescence and Confocal Imaging

Freshly dissected cochlear basilar membrane whole mounts were used for hair cell and ribbon synapse staining. Samples were incubated overnight at 4°C with anti‐Myosin VIIa (Proteus, #25‐6790, 1:200), anti‐CtBP2 (mouse IgG1, BD Biosciences, #612044, 1:500), and anti‐GluR2 (clone 6C4, mouse IgG2a, Millipore, #MAB3972, 1:200). For CtBP2/GluR2 dual labeling, samples were incubated with Alexa Fluor 568‐conjugated goat anti‐mouse IgG1 (Invitrogen, #A21134, 1:500) and Alexa Fluor 647‐conjugated goat anti‐mouse IgG2a (Invitrogen, #A21241, 1:500) for 2 h at room temperature. Frozen cochlear sections were prepared as previously described [61]. Samples were also incubated overnight at 4°C with primary antibodies. The antibodies and dilutions were anti‐Cntn2 (Abcam, #ab133498, 1:200), anti‐Cntn1 (R&D Systems, #AF904, 1:200), anti‐Kv1.2 (Alomone, #APC‐010, 1:100), anti‐NRCAM (Abcam, #ab24344, 1:200), and anti‐NF200 (Sigma–Aldrich, #N0142, 1:500). The next day tissues were washed with 1× PBS (0.01 m). They were then incubated with appropriate secondary antibodies. These included anti‐rabbit Alexa Fluor 488 (Invitrogen, #A11034, 1:500) and goat anti‐mouse Alexa 555 (Life Technologies, #37459, 1:500). Incubation was in blocking buffer for 2 h at room temperature. After washing with PBS, samples were counterstained. DAPI (Servicebio, #G1012, 1:1000) was applied for 15 min at room temperature. Alternatively, Alexa Fluor 568 phalloidin (Servicebio, #G1041, 1:200) was applied for 0.5 h at room temperature.

Mice were anesthetized, and brain tissues were quickly extracted. The tissues were fixed in 4% paraformaldehyde, dehydrated through a sucrose gradient, and embedded in OCT compound. Brain sections containing the cochlear nuclei and auditory cortex were collected according to the mouse brain atlas at 10‐µm slice thickness. Sections were washed with 1× PBS, blocked with 10% bovine serum albumin, and permeabilized using 0.1% Tween‐20. Subsequently, five primary antibodies: Cntn1, Cntn2, Kv1.2, NRCAM, and NF200 were applied sequentially. After incubation with each primary antibody, the sections were washed with 1× PBS and incubated with the corresponding horseradish peroxidase–conjugated secondary antibodies (goat anti‐rabbit IgG, rabbit anti‐goat IgG, and goat anti‐mouse IgG). Signal amplification was performed using tyramide signal amplification reagents labeled with different fluorophores (iF440‐Tyramide, iF488‐Tyramide, iF546‐Tyramide, iF594‐Tyramide, and iF700‐Tyramide). After each antibody labeling step, sections were treated with pH 6.0 citrate buffer (#G1202, Servicebio), and microwave irradiation to remove bound antibodies and prevent cross‐reactivity. This process was repeated for labeling all five proteins. Finally, nuclei were counterstained with DAPI.

All samples were mounted using an anti‐fade medium (#ZG1028, VECTOR) and imaged using a confocal microscope (SP8, Leica, Germany). For ribbon synapse analysis, confocal Z‐stacks spanning the IHC synaptic region were acquired using a 63× oil‐immersion objective, 1.5× digital zoom, and a 0.3 µm Z‐step. The CtBP2 and GluR2 channels were examined separately and as merged images section by section throughout the Z‐stack using Leica imaging software. A morphologically intact ribbon synapse was defined as a CtBP2‐positive punctum juxtaposed to a GluR2‐positive punctum and was manually counted by an investigator blinded to genotype. Approximately 8–10 IHCs were analyzed per cochlear region from each mouse. Counts were expressed as paired CtBP2/GluR2 puncta per IHC and averaged within each region for each mouse.

### 4.5 TEM

Mouse cochleae were dissected, fixed, and decalcified as previously described [61]. The samples were then immersed in 1% osmium tetroxide for 2 h, dehydrated through a graded acetone series, and embedded in resin (Epon 812, #45345, Sigma–Aldrich, Germany). Semi‐thin sections were stained with toluidine blue (#89640, Sigma–Aldrich, Germany) for general structural assessment. Ultrathin sections were subsequently stained with alkaline lead citrate and uranyl acetate. Ultrastructural features of the cochleae were examined using a Hitachi HF5000 transmission electron microscope (Hitachi, Tokyo, Japan). For myelin morphometry, six cochleae per genotype were analyzed.

### 4.6 Transcriptome Analysis

Cochleae were harvested from 2‐month‐old WT and *Cntn2^+/−^* mice (n = 3 per group; 2 cochleae per sample). mRNA extraction and cDNA library construction were performed by OE Biotech (Shanghai, China). Differential expression analysis was conducted using DESeq2, with thresholds set at fold change > 1.5 or *p* < 0.05 to identify DEGs. DEG Enrichment analysis was performed based on hypergeometric distribution and GO and Kyoto Encyclopedia of Genes and Genomes pathway enrichment results were visualized using bubble plots. GSEA was performed using GSEA software to rank genes by the extent of differential expression and assess enrichment of predefined gene sets. For comparative analysis, DEGs in the ANs of young and aged mice were examined using publicly available transcriptomic datasets [29].

### 4.7 ROS Assay

To assess oxidative damage in the auditory pathway of *Cntn2^+/−^* mice, DHE staining, and 4‐HNE immunostaining were performed. Frozen sections of mouse cochlear and brain tissues were incubated with 1 mm DHE (#D23107, Thermo Fisher, USA) in PBS for 30 min at 37°C. For 4‐HNE detection, sections were incubated overnight at 4°C with an anti‐4‐HNE antibody (Alpha Diagnostic, #HNE11‐S, 1:100). The next day, tissues were washed with 1× PBS and incubated for 2 h at room temperature with an Alexa Fluor 488‐conjugated anti‐rabbit secondary antibody (1:500; Invitrogen, USA), followed by nuclear staining with DAPI (#G1012, Servicebio, China) for 15 min.

### 4.8 AAV Vector Preparation and In Vivo Delivery

Recombinant AAV9 vectors were generated by OBiO Technology (Shanghai, China). AAV9‐CMV‐Cntn2 carried full‐length mouse Cntn2 cDNA (NM_009396) driven by a CMV promoter with tWPA enhancer (plasmid H51794, 7069 bp). AAV9‐CMV‐Cntn2 contained no fluorescent reporter, as the AAV packaging capacity did not permit inclusion of both full‐length Cntn2 and EGFP. At P1, mice were anesthetized by brief hypothermia in an ice bath for 2–3 min until reflex responses were absent. Following a postauricular incision, the auditory bulla was exposed, and the round window membrane was visualized under a dissecting microscope. A glass micropipette with a 25 µm tip was inserted through the round window membrane into the scala tympani. The left cochlea received 0.5 µL AAV9‐CMV‐Cntn2 at 2.74 × 10^1^ ^3^ genomic copies/mL, delivered at 1 µL/min using an UMP3 microsyringe pump (World Precision Instruments). The right cochlea received an equal volume of empty AAV9 vector at the same viral titer using the same procedure and served as the paired vector control. The micropipette was retained in place for 2 min after infusion to minimize reflux before being withdrawn. A separate cohort received AAV9‐CMV‐EGFP in the left cochlea to evaluate cochlear transduction. The incision was sealed with veterinary tissue adhesive, and pups were returned to a 38°C heat pad for recovery. ABR were recorded 8 weeks post injection (2 months of age). Cochleae were subsequently harvested for histological and molecular analysis.

### 4.9 Western Blot Analysis

Cochlear auditory nerve, cochlear nucleus, and auditory cortex tissues were homogenized on ice in RIPA lysis buffer supplemented with protease inhibitors. Lysates were centrifuged at 12 000 × g for 15 min at 4°C, and protein concentrations in the supernatants were determined using a BCA protein assay. Equal amounts of protein (40 µg per lane) were separated on 8%–12% SDS‐PAGE gels according to the molecular weight of the target protein and transferred onto 0.45 µm PVDF membranes by wet transfer at 300 mA for 2 h at 4°C. Membranes were blocked with 5% nonfat milk in TBST for 1 h at room temperature and incubated overnight at 4°C with the following primary antibodies: anti‐CNTN2 (rabbit monoclonal, Abcam, #ab133498, 1:1000), anti‐CNTN1 (goat polyclonal, R&D Systems, #AF904, 1:1000), anti‐Kv1.2 (rabbit polyclonal, Alomone Labs, #APC‐010, 1:1000), anti‐NRCAM (rabbit polyclonal, Abcam, #ab24344, 1:1000), anti‐4‐HNE (rabbit polyclonal, Alpha Diagnostic International, #HNE11‐S, 1:1000), and anti‐GAPDH (mouse monoclonal, Beyotime, #AF0006, 1:1000). After three washes with TBST, membranes were incubated for 1 h at room temperature with the appropriate HRP‐conjugated secondary antibodies: goat anti‐rabbit IgG (Beyotime, #A0208, 1:2000), donkey anti‐goat IgG (Beyotime, #A0181, 1:1000), or goat anti‐mouse IgG (Beyotime, #A0216, 1:1000). Immunoreactive bands were visualized using enhanced chemiluminescence and quantified using ImageJ. Signals corresponding to CNTN2, CNTN1, Kv1.2, and NRCAM were quantified at their expected molecular‐weight regions and normalized to GAPDH. For 4‐HNE analysis, all detectable immunoreactive bands between 20 and 150 kDa were integrated after background subtraction and normalized to GAPDH.

### 4.10 Statistical Analysis

Data were analyzed using GraphPad Prism 10.0 software and are presented as mean ± standard deviation (SD). Comparisons between two independent groups were performed using two‐sided unpaired Student's t‐tests. Paired comparisons between the AAV9‐CMV‐Cntn2‐treated left ear and the empty‐vector‐treated right ear from the same mouse were performed using two‐sided paired Student's t‐tests. ABR and DPOAE measurements across frequencies or stimulus intensities were analyzed using two‐way repeated‐measures ANOVA followed by Bonferroni's multiple‐comparisons test. Measurements across apical, middle, and basal cochlear regions, including hair cell survival, and paired CtBP2/GluR2 puncta per IHC, were analyzed using two‐way repeated‐measures ANOVA followed by Bonferroni's multiple‐comparisons test. For the AAV experiment, ABR measurements from the treated and contralateral control ears were analyzed using two‐way repeated‐measures ANOVA followed by Bonferroni's multiple‐comparisons test, whereas Western blot measurements were analyzed using a two‐sided paired Student's t‐test. Statistical significance was defined as *p* < 0.05. ns, *p* > 0.05; *p < 0.05; **p < 0.01; ***p < 0.001.

## Conflicts of Interest

The authors declare no conflicts of interest.

## Supporting information




**Supporting File 1**: advs78462‐sup‐0001‐SuppMat.docx.

**Supporting File 2**: advs78462‐sup‐0002‐Data.zip.

## Data Availability

The data is available from supplementary material and the corresponding author on reasonable request.
