# Supplementary material for: CNTN2 Deficiency Disrupts Ascending Auditory Neuroglial Interactions and Redox Homeostasis in Neural Presbycusis
Source: Adv Sci (Weinh). 2026 Oct 26:e22800. Online ahead of print. doi: 10.1002/advs.202522800 (PMC13710709; doi:10.1002/advs.202522800)
Supplement: Supplementary file 1 [file ADVS-9999-e22800-s001.docx]

**Supporting Information**

**CNTN2 Deficiency Disrupts Auditory Neuroglial Integrity and Redox Homeostasis in Neural Presbycusis**

Penghui Chena,b,1, Jingyi Zhua,c,1, Sihan Huanga,c,1, Beibei Xu a,c, Maoli Duan^d, e^, Jingrong Lv a,c,*, Jiarui Chen^b,*^, Jun Yang a,c,*, Shule Hou a,c,*

*This Supporting Information file contains Supplementary Figures S1–S8. The individual numerical values underlying all bar graphs, scatter plots, and ABR input-output functions are provided in the Source Data file (Data S1.xlsx).*

**Supplementary Figures**

**Figure S1. Generation and validation of the Cntn2 heterozygous knockout mouse model.**

(A) Schematic of the CRISPR/Cas9-mediated targeting strategy. A Cre-EGFP-SV40 polyA cassette was inserted into exon 2 of the Cntn2 gene, replacing the ATG start codon to achieve heterozygous knockdown (*Cntn2^+/-^*). SPF/SPR: single-guide RNA target sites; yellow: knock-in region. (B, C) Western blot verification of CNTN2 knockdown efficiency. CNTN2 protein levels are reduced by approximately 50% in *Cntn2^+/-^* mice compared to WT littermates. ***P < 0.001. (D) Immunofluorescence staining of CNTN2 (red) in spiral ganglion neurons confirming reduced protein expression in *Cntn2^+/-^* mice. Blue: DAPI. Scale bar: 20 μm. *Cntn2^+/-^*, red: CNTN2, blue: DAPI, *** *P* < 0.001, Scale bar: 20 μm.

**Figure S2. Schematic overview of the experimental design.**

The study included aging analysis, auditory phenotyping of Cntn2+/− mice, examination of cochlear synapses and auditory nerve pathology, analysis of nodal proteins and oxidative stress along the auditory pathway, and early AAV-mediated CNTN2 supplementation. Auditory and molecular outcomes were assessed at 2 months of age.

**Figure S3. Cross strain validation and age-related auditory phenotypes in C57BL/6J mice.**

(**A**) ABR thresholds in 2-month-old C57BL/6J (n = 6) and CBA/CaJ (n = 6) mice showing no significant differences across frequencies. (**B, C**) 16 kHz wave I amplitude and latency were comparable between the two strains at 2 months of age (ns). (**D**) ABR thresholds in young (2-month, n = 6) and aged (12month, n = 6) C57BL/6J mice. Aged C57 mice exhibited elevated thresholds at high frequencies. (**E, F**) 16 kHz wave I and II amplitudes were reduced in aged C57 mice. (**G, H**) 16 kHz wave I and II latencies were prolonged in aged C57 mice. (**I**) 16 kHz interpeak interval (II-I) showed minimal change in aged C57BL/6J mice compared to young controls. Data are mean ± SD; ns P > 0.05, *P < 0.05, **P < 0.01, ***P < 0.001; two-way ANOVA with Sidak’s multiple comparisons test.

**Figure S4. Detailed 11 kHz and 32 kHz ABR analysis in *Cntn2^+/−^* mice and littermate WT controls.**

**(A-E)** Amplitude intensity functions for waves I-V at 32 kHz. **(F-J)** Latency intensity functions for waves I-V at 32 kHz. **(K-O)** Interpeak intervals (IPIs) for waves II-I (K), III-I (L), IV-I (M), V-I (N), and V-III (O) at 32 kHz. Black: WT; red: *Cntn2^+/-^*. At 32 kHz, *Cntn2^+/-^* mice exhibit prolonged latencies and increased IPIs (II-I, III-I, IV-I, V-I), while amplitude differences are more variable. ns *P* > 0.05, **P* < 0.05, ***P* < 0.01; two-way ANOVA with Sidak’s multiple comparisons test; *n* = 6-7 mice per group.

**Figure S5. Myelin thickness and lamellar number in auditory nerve fibers of Cntn2-deficient mice.**

**(A, B)** Representative transverse TEM images of myelinated auditory nerve fibers from 2-month-old *Cntn2^+/−^* **(A)** and WT **(B)** mice. Boxed regions are shown at higher magnification. Focal myelin outfolding and lamellar disorganization are visible in the *Cntn2^+/−^* sample, whereas the WT myelin sheath shows a compact and regularly arranged lamellar structure. Scale bars: 0.5 μm in the low-magnification images and 200 nm in the enlarged images. **(C, D)** Quantification of myelin lamellar number per fiber **(C)** and myelin thickness **(D)**. Each point represents the mean value for one mouse. Data are presented as mean ± SD; n = 6 cochlea per genotype; two-sided unpaired Student’s t-test; ns, *P* > 0.05.

**Figure S6. Western blot validation of nodal protein expression in the cochlea.**

**(A)** Representative Western blot images of CNTN2, CNTN1, Kv1.2, and NRCAM in cochlear auditory nerve lysates from *Cntn2^+/-^* and WT mice. **(B-E)** Quantification of band intensities normalized to GAPDH. All four proteins show significant reduction in *Cntn2^+/-^* mice. Data are mean ± SD; **P* < 0.05, ****P* < 0.001; Student’s two-tailed *t*-test; *n* = 3 mice per group.

**Figure S7. Western blot validation of nodal protein expression in central auditory structures.**

**(A)** Representative Western blot images of CNTN2, CNTN1, Kv1.2, and NRCAM in the cochlear nucleus (CN) and auditory cortex (AC) of *Cntn2^+/-^* and WT mice. **(B-E)** Quantification normalized to GAPDH. All proteins are significantly reduced in both CN and AC of *Cntn2^+/-^* mice. Data are mean ± SD; **P* < 0.05, ***P* < 0.01; Student’s two-tailed *t*-test; *n* = 3 mice per group.

**Figure S8. Western blot validation of oxidative stress markers across the auditory pathway.**

**(A)** Representative Western blot images of 4-hydroxynonenal (4-HNE) protein adducts in the cochlea, cochlear nucleus (CN), and auditory cortex (AC) of *Cntn2^+/-^* and WT mice. **(B)** Quantification of 4-HNE band intensities normalized to GAPDH. 4-HNE is significantly elevated across all three auditory levels in *Cntn2^+/-^* mice. Data are mean ± SD; **P* < 0.05, ***P* < 0.01; Student’s two-tailed *t*-test; *n* = 3 mice per group.
